# Supplementary figures and images for: Regulation of bacterial surface attachment by a network of sensory transduction proteins
Source: PLoS Genet. 2019 May 10;15(5):e1008022. doi: 10.1371/journal.pgen.1008022 (PMC6530869; doi:10.1371/journal.pgen.1008022)

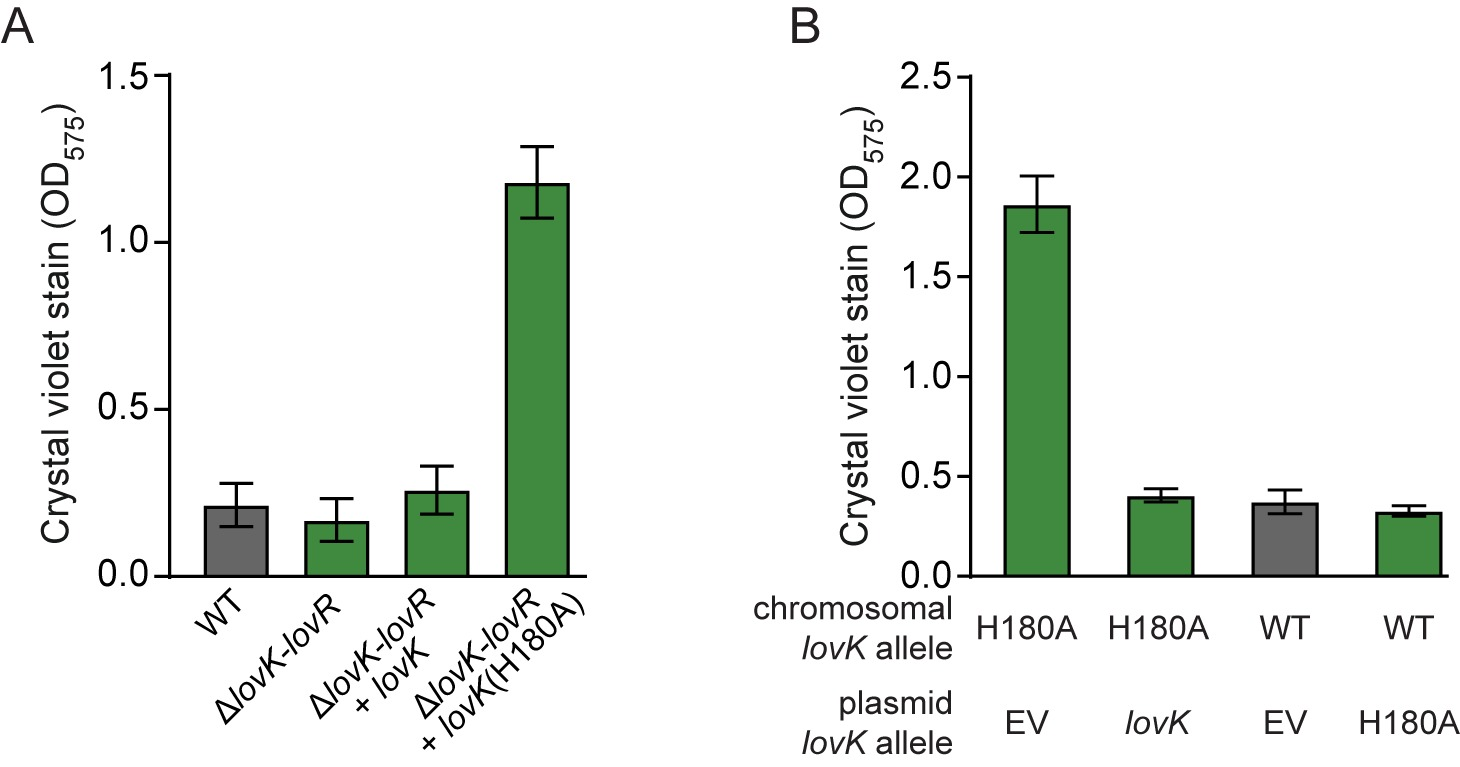

Supplement: S1 Fig — A) Strains bearing a deletion of the lovK-lovR locus were complemented with a wild-type lovK or lovK(H180A) allele. Surface attachment to polystyrene plates was measured by crystal violet stain. Cultures were grown in M2X medium. Data are representative of at least three independent experiments. Bars represent mean ± s.d.; n = 7. B) Surface adhesion (crystal violet stain) measured in different genetic backgrounds. Cultures were grown in M2X medium. Data are representative of at least three independent experiments. Bars represent mean ± s.d.; n = 8. (TIF) [file pgen.1008022.s001.tif]

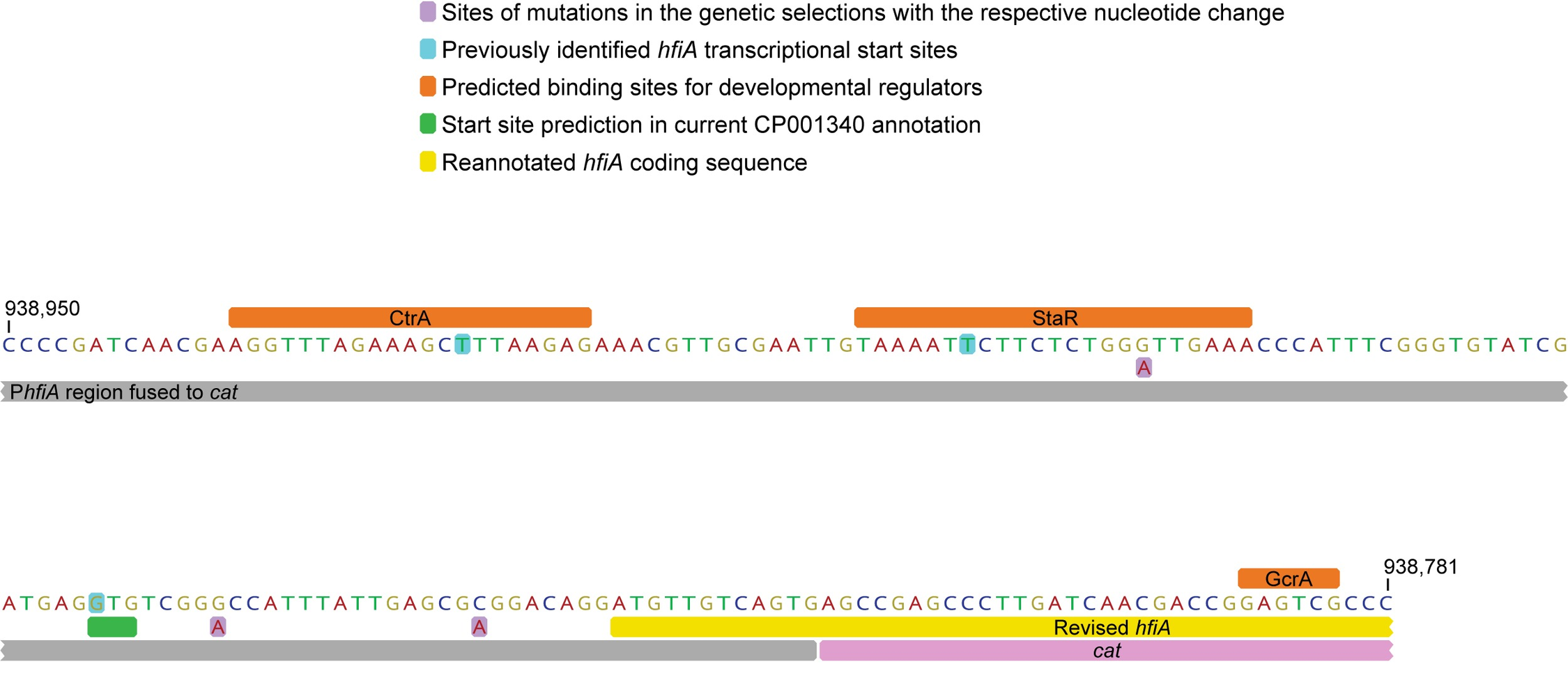

Supplement: S2 Fig — Predicted DNA-binding regions of several developmental regulators in orange [14]. Predicted start codon corresponding to original hfiA annotation in green; the revised hfiA reading frame is in yellow [14]. Experimentally identified hfiA transcriptional start sites in light blue [14] [74]. The central portion of the PhfiA-cat fusion is shown with PhfiA in gray while and cat in pink. Sites of spontaneous mutations identified in the genetic selections with the respective nucleotide change in light purple. Genome coordinates are based on the NA1000 genome sequence (CP001340; NC_011916). (TIF) [file pgen.1008022.s002.tif]

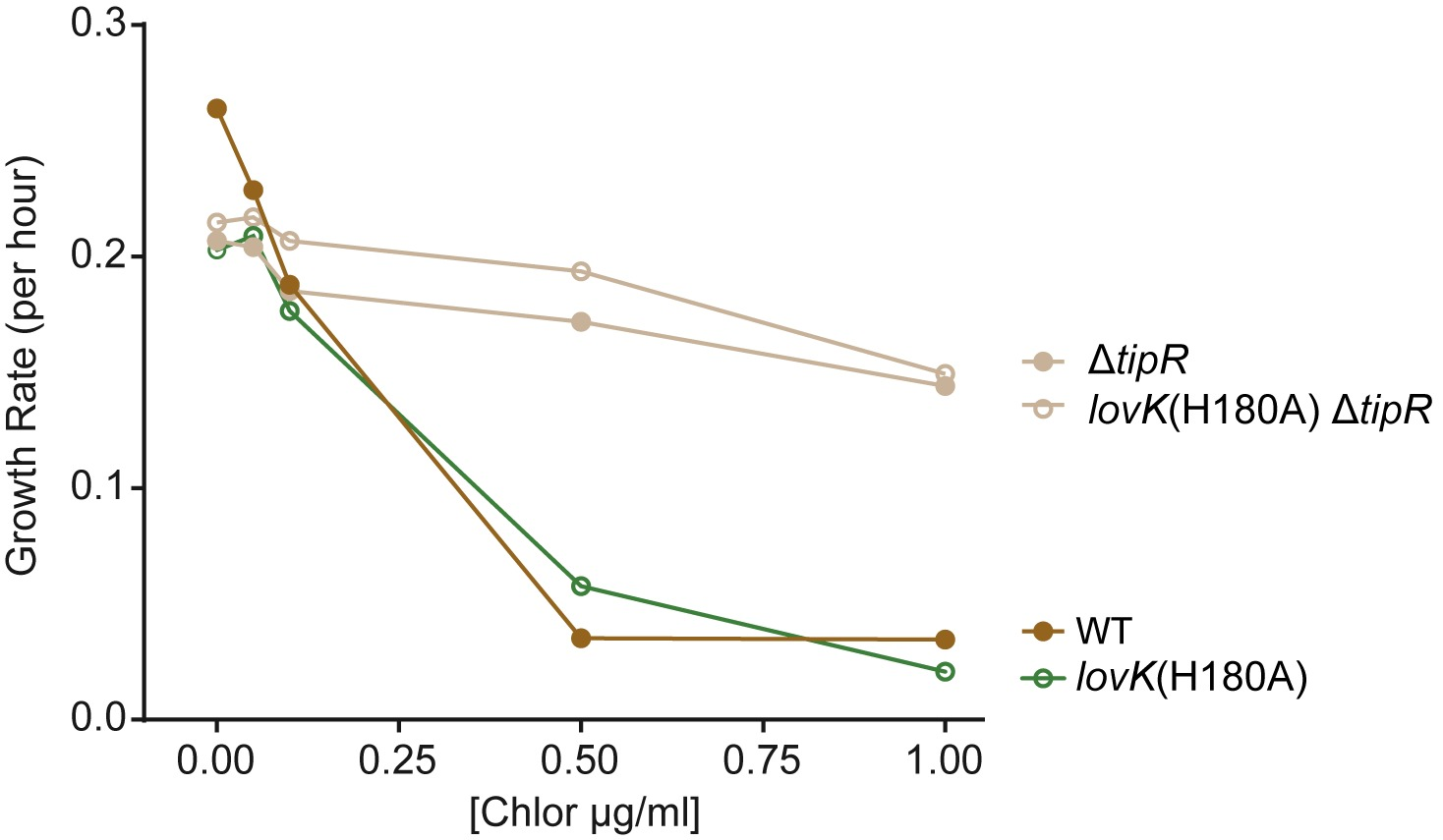

Supplement: S3 Fig — Growth rate per hour at different chloramphenicol concentrations for wild-type (WT), lovK(H180A), ΔtipR, and lovK(H180A) ΔtipR strains. Cultures were grown in M2X medium supplemented to the final chloramphenicol concentrations indicated on the x-axis. One biological replicate per condition per strain. (TIF) [file pgen.1008022.s003.tif]

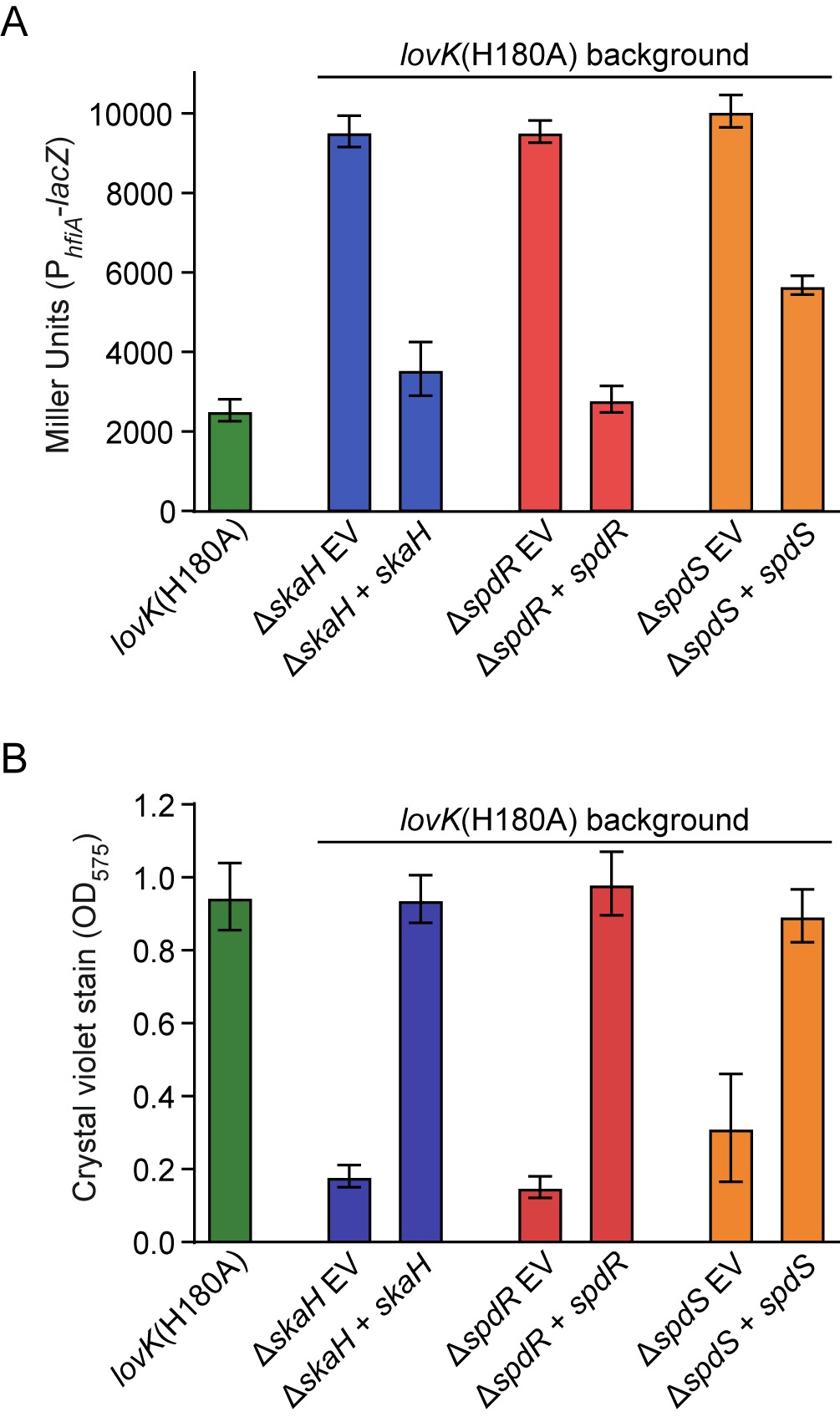

Supplement: S4 Fig — Strains bearing in-frame deletions of skaH, spdS or spdR in a lovK(H180A) background were transformed with either pMT680 plasmids that express the corresponding disrupted gene from a xylose inducible promoter or pMT680 as an empty vector (EV) control. A) β-galactosidase activity from the PhfiA-lacZ transcriptional fusion was measured in each strain. Cultures were grown in M2X medium to OD660nm of 0.05–0.15. Presented data are representative of at least three independent experiments. Bars represent mean ± s.d.; n = 6. B) Surface attachment of cells grown in polystyrene plates was measured by crystal violet stain. Cultures were grown in M2X medium for 16 hrs post-inoculation. Data are representative of three independent experiments. Bars represent mean ± s.d.; n = 9. (TIF) [file pgen.1008022.s004.tif]

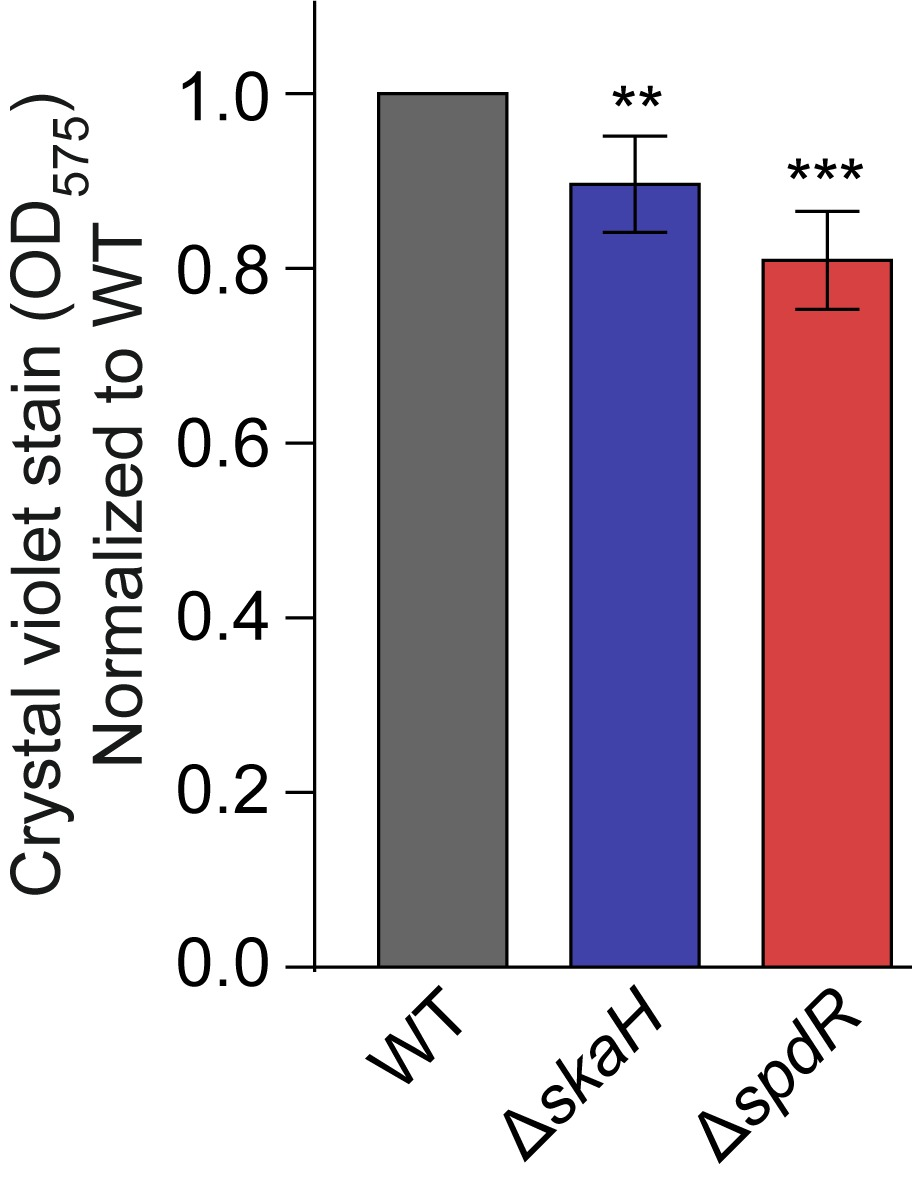

Supplement: S5 Fig — Crystal violet stain from cells growing in polystyrene plates from seven different experiments was collected. The mean ΔskaH and ΔspdR crystal violet stain for each experiment was normalized to the mean wild-type stain of the respective day. One-way ANOVA followed by Dunnett’s multiple comparisons test was performed using GraphPad Prism version 8.0.0. to compare wild-type to the mutant strains. ** P<0.005, *** P<0.0005. (TIF) [file pgen.1008022.s005.tif]

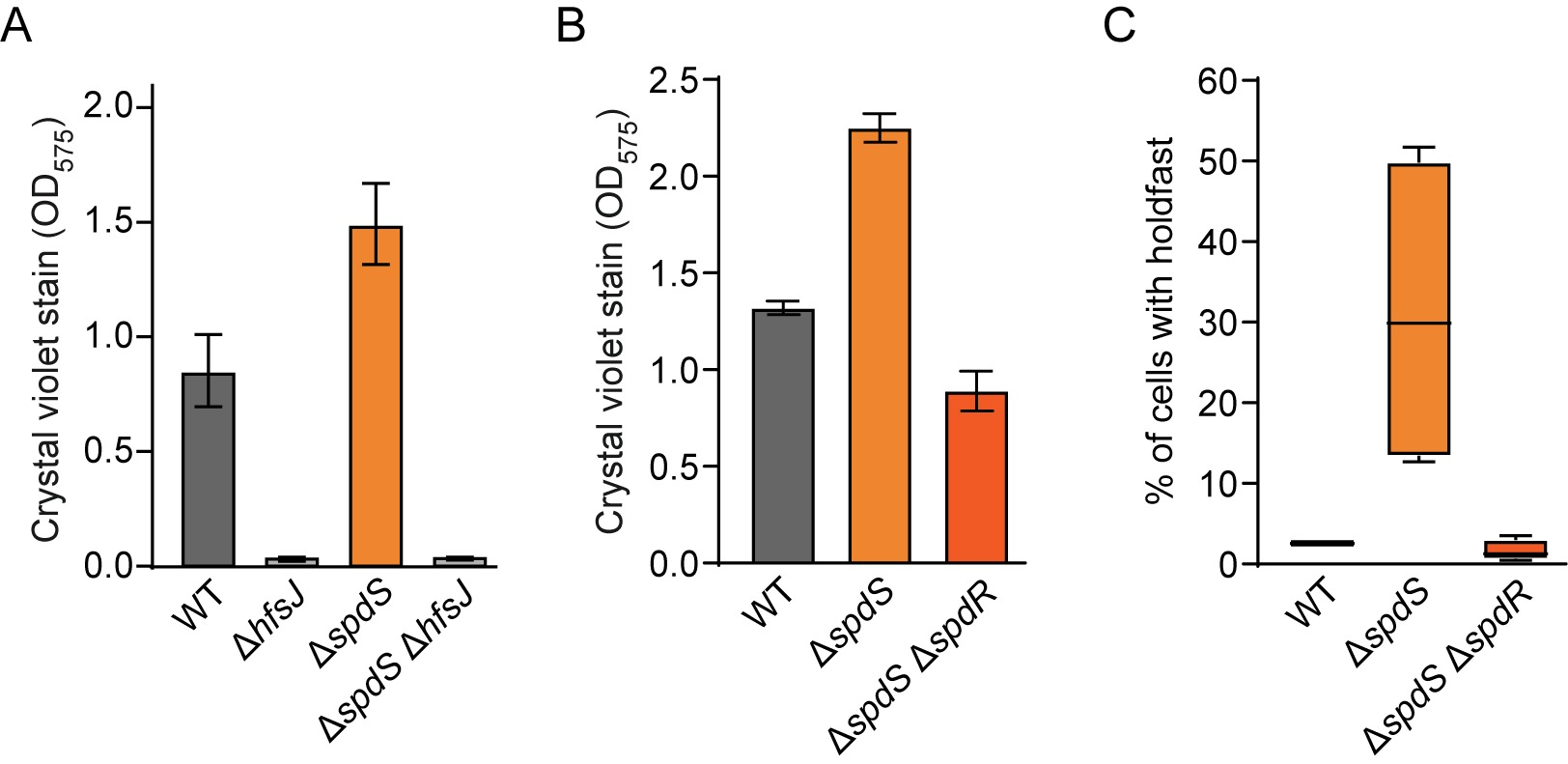

Supplement: S6 Fig — A) Surface attachment to polystyrene plates was measured by crystal violet stain in wild-type (WT), ΔspdS, ΔhfsJ and ΔspdSΔhfsJ strains. Cultures were grown in M2X medium until stationary phase (24 hrs post-inoculation). Data are representative of at least three independent experiments. Bars represent mean ± s.d.; n = 12. B) Surface attachment to polystyrene plates was measured by crystal violet stain in wild-type (WT), ΔspdS, and ΔspdSΔspdR strains. Cultures were grown in M2X medium until stationary phase (24 hrs post-inoculation). Data are representative of at least three independent experiments. Bars represent mean ± s.d.; n = 12. C) Holdfast was stained with fluorescent Wheat Germ Agglutinin in wild-type (WT), ΔspdS, and ΔspdSΔspdR strains. Percentage of cells bearing a holdfast was quantified by microscopy. Cultures were grown in polystyrene plates with M2X medium until stationary phase (24 hrs post-inoculation). Bars represent mean ± s.d.; n = 4. (TIF) [file pgen.1008022.s006.tif]

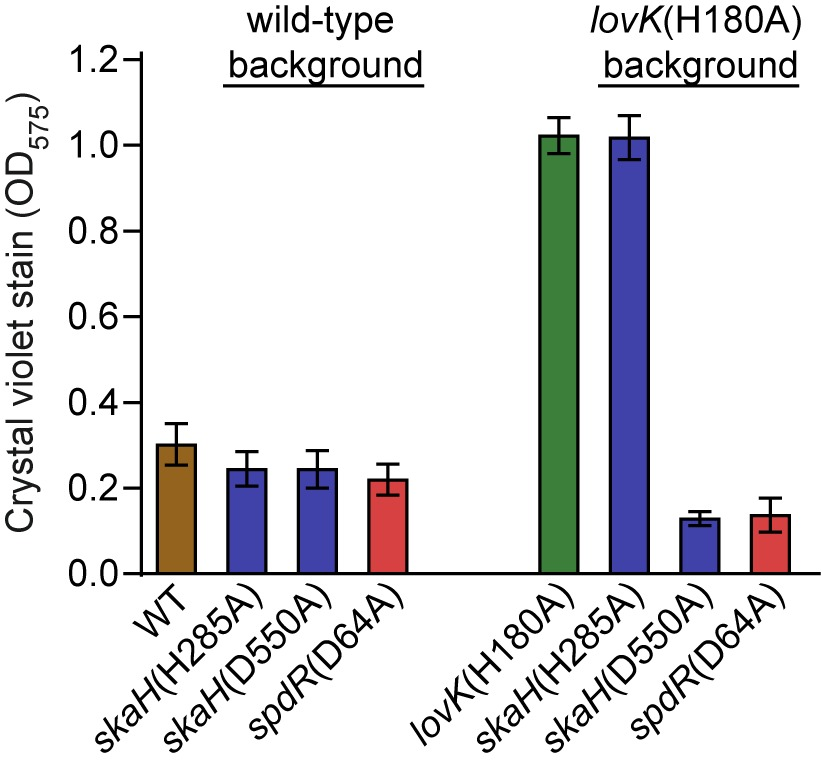

Supplement: S7 Fig — Strains bearing point mutations in the conserved phosphorylation sites of skaH, spdS or spdR in either a wild-type (WT) or lovK(H180A) background were grown in M2X medium in polystyrene plates for 16 hrs post inoculation. Surface attachment was measured by crystal violet stain. Data presented are representative of three independent experiments. Bars represent mean ± s.d.; n = 8. (TIF) [file pgen.1008022.s007.tif]

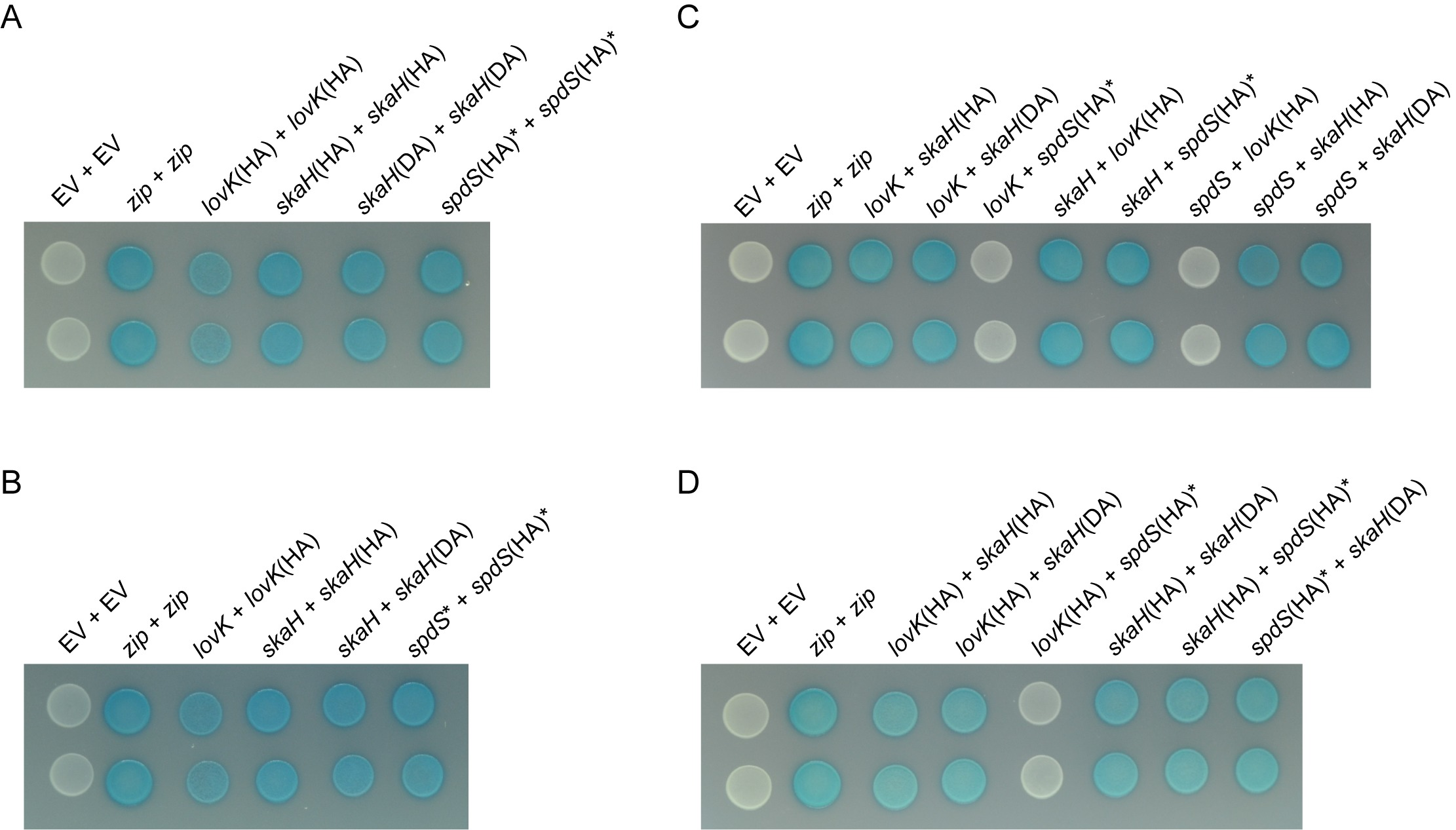

Supplement: S8 Fig — A-D) Bacterial two-hybrid (BTH) experiments to assess interactions between mutated histidine kinase fusions to a split adenylate cyclase. lovK(HA) = lovK(H180A), skaH(HA) = skaH(H285A), skaH(DA) = skaH(D550A), spdS(HA)* = spdS(H248A)*. Fusions with spdS lack the transmembrane domain, notated spdS* or spdS(HA)*. Zip = positive control. Two biological replicates are shown for each co-expression combination. Protein-protein interaction of fusions reconstitutes the split adenylate cyclase encoded on pKT25 and pUT18c, and results in a blue color on agar plates containing x-gal. Strains expressing fusions that do not interact appear white. (TIF) [file pgen.1008022.s008.tif]

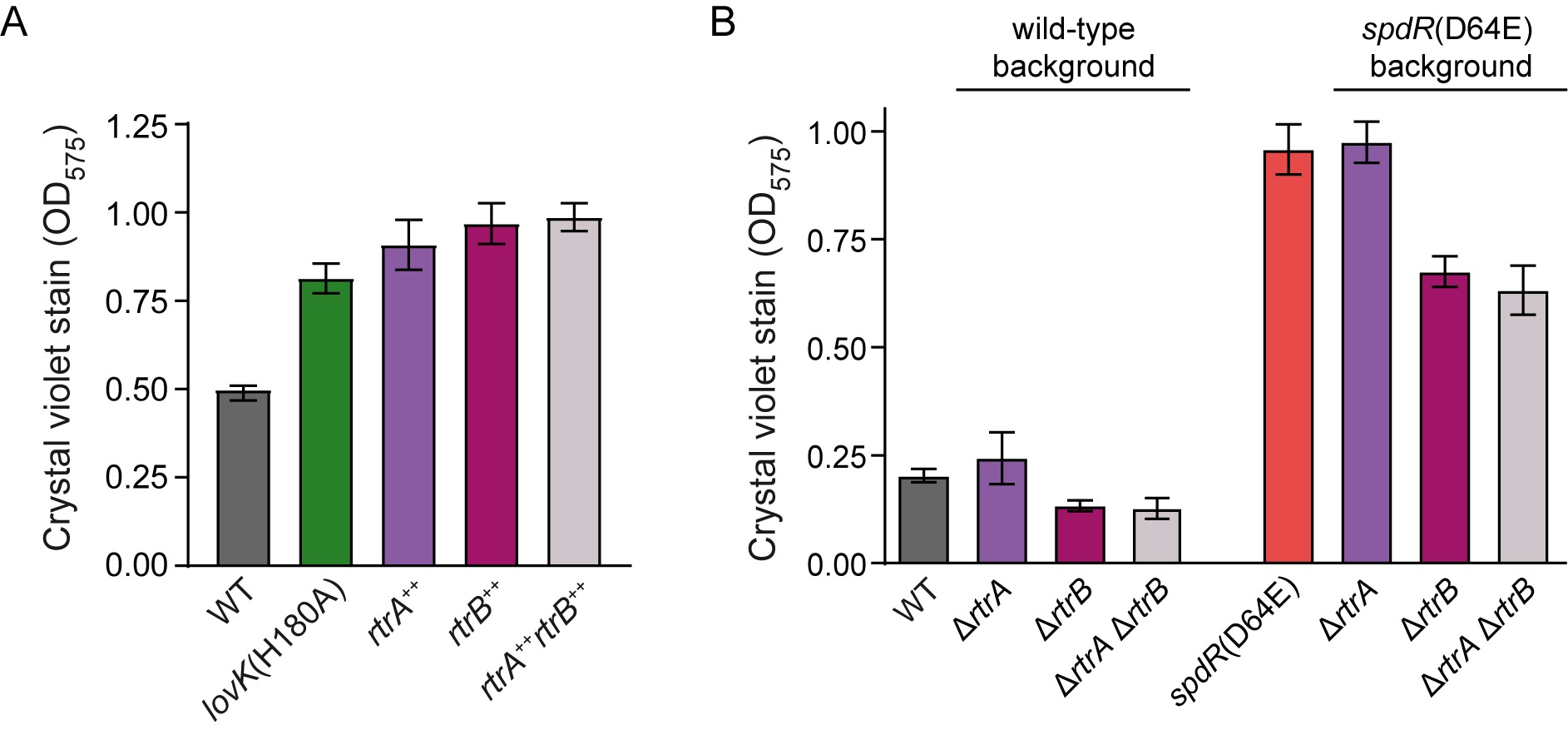

Supplement: S9 Fig — A) Surface attachment of cells grown in polystyrene plates was measured by crystal violet stain. Wild-type (WT) and lovK(H180A) carry empty vectors of pMT680 and pMT585. rtrA++ carries pMT680-rtrA and pMT585 empty vector. rtrB++ carries pMT585-rtrB and pMT680 empty vector. rtrA++rtrB++ carries pMT680-rtrA and pMT585-rtrB. Cultures were grown for 16 hrs post-inoculation in M2X medium. Data presented are representative of at least three independent experiments. Data represent mean ± s.d.; n = 6–8. B) Surface attachment of cells grown in polystyrene plates was measured by crystal violet stain in ΔrtrA, ΔrtrB, and ΔrtrAΔrtrB in a wild-type (WT) and spdR(D64E) backgrounds. Cultures were grown for 16 hrs post-inoculation in M2X medium. Data presented are representative of at least three independent experiments. Data represent mean ± s.d.; n = 9. (TIF) [file pgen.1008022.s009.tif]

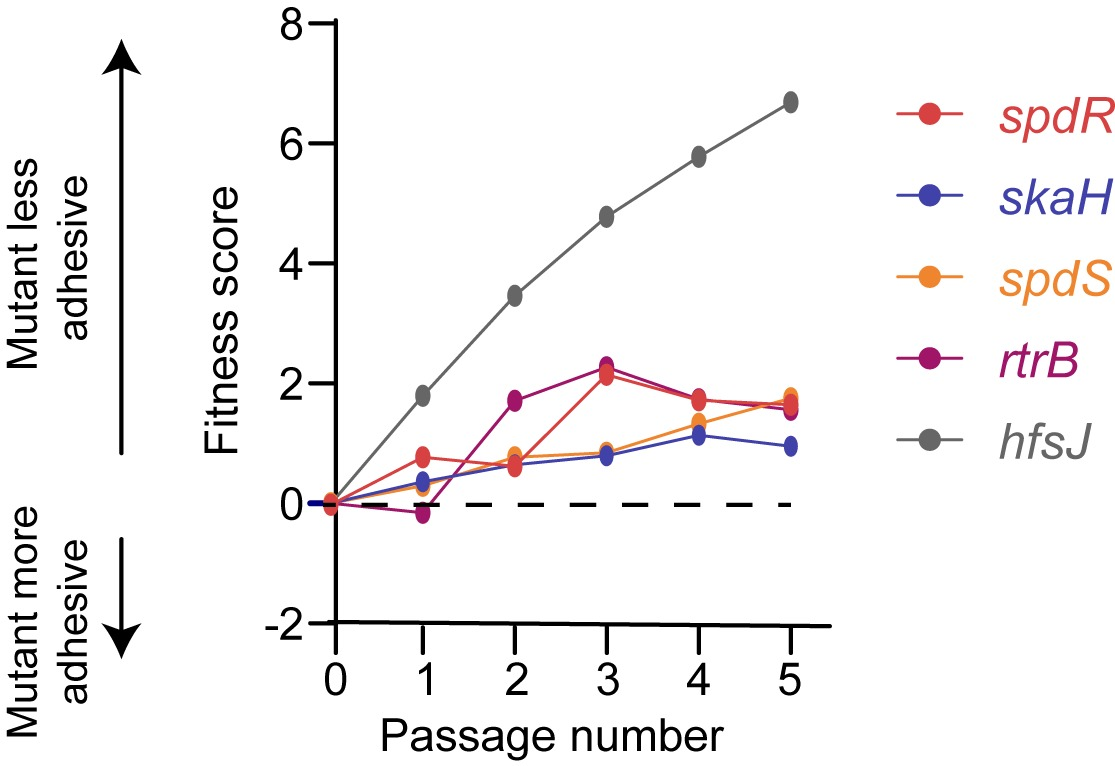

Supplement: S10 Fig — Extracted fitness profiles of mutants characterized in a genome-wide attachment screen [58]. Briefly, a C. crescentus transposon mutant library was grown and passaged in complex PYE medium in the presence of cheesecloth to provide surface area for cell attachment. Cells were periodically sampled from the broth over several days. Hyper-adhesive mutants are titrated out of the broth by the cheesecloth and, consequently, have negative fitness scores (in which the fitness score is a log2 ratio of mutant strain abundance relative to the average strain). Conversely, hypo-adhesive mutants are enriched in the broth resulting in positive fitness scores. Strains bearing transposon insertions in TCS genes identified in our study have positive fitness scores. hfsJ mutants, which do not produce holdfast and are non-adhesive, are presented as a reference. rtrA was not captured in this analysis; the library contained insufficient Tn-insertions in this gene. (TIF) [file pgen.1008022.s010.tif]
